# Angular engineering strategy of an additional periodic phase for widely tunable phase-matched deep-ultraviolet second harmonic generation

**DOI:** 10.1038/s41377-022-00715-w

**Published:** 2022-02-04

**Authors:** Mingchuan Shao, Fei Liang, Haohai Yu, Huaijin Zhang

**Affiliations:** State Key Laboratory of Crystal Materials and Institute of Crystal Materials, Shandong University, Jinan, 250100 China

**Keywords:** Nonlinear optics, Optical physics

## Abstract

Manipulation of the light phase lies at the heart of the investigation of light-matter interactions, especially for efficient nonlinear optical processes. Here, we originally propose the angular engineering strategy of the additional periodic phase (APP) for realization of tunable phase matching and experimentally demonstrate the widely tunable phase-matched second harmonic generation (SHG) which is expected for dozens of years. With an APP quartz crystal, the phase difference between the fundamental and frequency-doubled light is continuously angularly compensated under this strategy, which results the unprecedented and efficient frequency doubling at wavelengths almost covering the deep-UV spectral range from 221 to 332 nm. What’s more, all the possible phase-matching types are originally realized simultaneously under the angular engineering phase-matching conditions. This work should not only provide a novel and practical nonlinear photonic device for tunable deep-UV radiation but also be helpful for further study of the light-matter interaction process.

## Abstract (web summary)

A widely tunable phase-matched second harmonic generation (221–332 nm) covering almost the entire deep-UV spectral range is experimentally realized by angular engineering strategy of an additional periodic phase.

## Graphical abstract

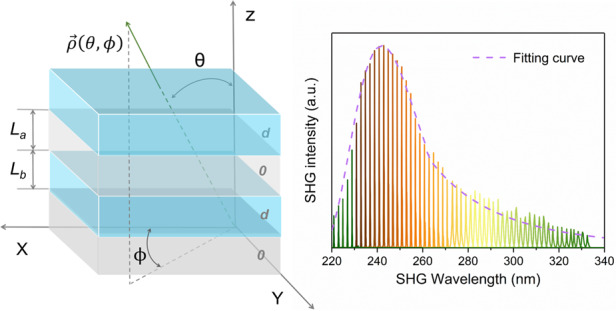

## Introduction

Phase is a “memory” of motion in the classical and quantum wave functions^1–3^ and can record the trajectory and states of particles and waves in time and space simultaneously^4,5^. Phase engineering of a wave function influences and determines the tendency and even results of physical processes, which could also give rise to abundant fascinating phenomena, such as the soliton, Moiré lattices and topological charge^6–8^, thus boosting the development of integrated photonics, phononics and electronics^9–11^. For the electromagnetic wave, its behaviour can be well described by Maxwell’s equation, and manipulation of the phase results in weak-field linear optics, including reflection, refraction, scattering and interferometric control of absorption^12–15^. Under strong electromagnetic fields with ultrahigh electric field intensity, the interacting matter could respond nonlinearly and generate nonlinear polarization and nonlinear optics^16^, whose efficiency also depends on the phase relationship, with manifestation of momentum conservation. For example, the energy flow could be inverted when the phase difference reaches π in the second harmonic generation (SHG) process if the phase is mismatched. To realize highly efficient optical conversion, a phase-matching condition between the fundamental and harmonic electromagnetic waves is required. This prerequisite was realized in many anisotropic crystals with inherent birefringence, which broadens the available wavelength of laser sources^17,18^ but concurrently precludes many nonlinear materials without suitable birefringence. To break the strict birefringent limitation, the quasi-phase-matching (QPM) technique was proposed in 1962 by employing periodic inversion of the polarization direction of nonlinear crystals^19^, which has been demonstrated in many ferroelectric materials^20–22^ and applied in classical and quantum optics fields for the generation of frequency conversion, nonlinear imaging, quantum information generation and computation^23–25^. However, there are also some “clouds” in phase-matching nonlinear optics, e.g., crystals without suitable birefringence and invertible ferroelectric domains were ruled out, which constrains the further development and application of nonlinear photonics in the broad spectral range, especially in the ultraviolet (UV) region. In addition, the well-designed frequency converters are wavelength- and polarization-dependent, which means that the typical wavelength requires a particular frequency converter with particular phase-matching type. The novel strategy for the efficient tunable frequency conversion and universal nonlinear crystals are expected for dozens of years.

Recently, we proposed a novel and universal phase-matching strategy associated with phase manipulation^26^, named as additional periodic phase (APP) phase matching. Such phase-matching condition is attributed to the artificial manipulation of the optical phase in the periodic ordered/disordered alignments. Meanwhile, the light phase is related to its propagation length in the crystal, thereby giving rise to the possibility of the generation of tunable phase matching if the phase could be angularly engineered in angular APP phase matching, especially in the deep-UV region where the practical nonlinear optical crystals are rare and the available laser wavelengths are discrete up to now. The angular APP phase matching would provide a new way for the development and design of nonlinear optical materials without suitable birefringence but with excellent nonlinear optical properties (e.g., short UV cut-off edge or large nonlinear coefficient) in the deep-UV region.

## Results

Herein, we originally theoretically investigated angular engineering of the additional periodic phase in APP phase matching and experimentally realized efficient tunable SHG (221–332 nm) covering almost the entire deep-UV spectral range with a tunable range of 111 nm for the first time to our knowledge. In addition, all possible phase-matching types are also originally simultaneously achieved with the participation of phase variation induced by the distribution of the grating period within a certain range. This novel and practicable work should be further development of APP phase matching and could inspire further studies in nonlinear optics, photonics and even physics.

Under the plane-wave approximation, electric field with a frequency of *ω*_*l*_ (*l* = 1,2,3) and an interaction length of *z* can be expressed as^16^

1 $${\vec{E}} (\omega _l) = {\vec{E}} _le^{ - i(\omega _lt - k_lz + \varphi _0^l)} = {\vec{E}} _le^{ - i\varphi _l}$$

where $\varphi _l = \omega _lt - k_lz + \varphi _0^l$ (*l* = 1,2,3) are the phases of the mixed three waves corresponding to the wave vectors *k*_*l*_ (*l* = 1,2,3), and $\varphi _0^l$ (*l* = 1,2,3) are the initial phases of the three interacting waves. Considering three-wave mixing, the nonlinear coupled wave equation for the generation of harmonic wave ${\vec {{E}}_3}$ can be shown as

2 $$\frac{{d{\vec {{E}}_{_3}} }}{{d\varphi }} = \frac{{i\omega _3d_{{\mathrm{eff}}}}}{{cn(\omega _3)}}{{\vec{E}}_{1}} {{\vec{E}}_2} e^{ - i(\varphi _3 - \varphi _1 - \varphi _2)} = \frac{{i\omega _3d_{{\mathrm{eff}}}}}{{cn(\omega _3)}}{{\vec{E}}_1} {\vec{{E}}_2} e^{ - i\Delta \varphi }$$

where *d*_eff_ is the effective second-order nonlinear coefficient of the crystal, *c* represents the light velocity and *n* ($\omega _3$) is the refractive index of the light with a frequency of $\omega _3$. The phase difference of the three mixed waves can be expressed as

3 $$\begin{array}{l}\Delta \varphi = \varphi _3 - \varphi _1 - \varphi _2\\ \quad\,\,\,\, = (\omega _3 - \omega _2 - \omega _1)t - (k_3 - k_2 - k_1)z + \varphi _0^3 - \varphi _0^2 - \varphi _0^1\end{array}$$

**Fig. 1:**
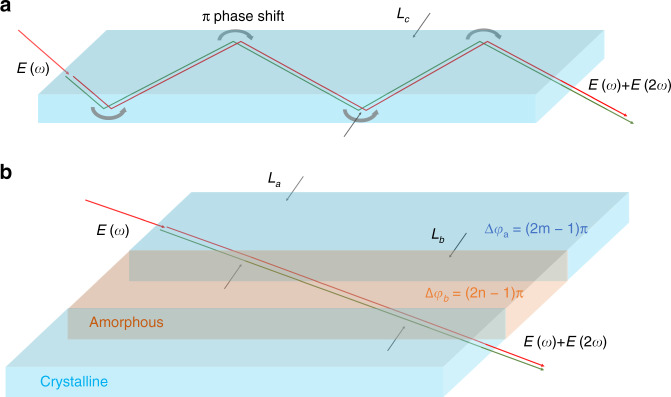
Two different ways to provide phase manipulation if the phase is mismatched. **a** Schematic of the traditional method with a $\pi$ phase shift at the interfaces. The interacting waves are totally reflected repeatedly with a propagation distance of $L_c = \frac{\pi }{{\Delta k}}$. Upon each reflection, $E\left( \omega \right)$ and $E\left( {2\omega } \right)$ are inverted with a $\pi$ phase shift at the interfaces. **b** Schematic of the APP with a continuous phase change. In crystalline regions, a nonlinear optical process occurs with a phase difference $\Delta \varphi _a = (2m - 1)\pi$, and then, in amorphous regions, phase manipulation operates with an APP $\Delta \varphi _b = (2n - 1)\pi$

During the optical conversion process, the conservation of energy requires $\omega _3 - \omega _2 - \omega _1 = 0$. The phase relationship should be dependent on $\Delta \varphi = \varphi _0^3 - \varphi _0^2 - \varphi _0^1 - (k_3 - k_2 - k_1)z$, which also provides parameters for manipulating the optical conversion efficiency. For $\Delta \varphi = 0$ and initial phase $\varphi _0^l = 0$, $\Delta k = k_3 - k_1 - k_2$ determines the optical conversion efficiency, with the principle that the energy can flow from the fundamental to harmonic waves in the coherence length $L_c = \frac{\pi }{{\Delta k}}$ with the phase difference $\Delta \varphi = {\uppi}$, and this above process is periodically reversed except under the condition of $\Delta k = 0$. In 1962, Armstrong et al. proposed that if the interacting waves could be totally reflected after a propagation distance of $L_c = \frac{\pi }{{\Delta k}}$, then the phase shift of π at the interfaces could compensate for the phase mismatch of π, as shown in Fig. 1a, which also derives the QPM conditions^18^.

Associated with the Bloch theory in condensed matter, the wave function $E(\varphi )$ of light with phase *φ* can be expressed as $E(\varphi ) = E(\varphi + 2N\pi )$, which means that the phase-matching conditions can be enlarged as $\Delta \varphi = 2N\pi$ with an integer *N*. The above discussion provides the possibility of “phase” matching instead of the strict birefringent phase-matching (BPM) and QPM conditions. Therefore, APP phase matching was proposed based on periodic phase manipulation, as shown in Fig. 1b, which was realized by periodic crystalline/amorphous alignments. By periodic division of *L*_*a*_ and *L*_*b*_, ordered regions with a length of *L*_*a*_ were employed for frequency conversion with $\Delta \varphi _a = \Delta kL_a = (2m - 1)\pi$, and disordered regions with a length of *L*_*b*_ were used for periodic phase compensation with $\Delta \varphi _b = (2n - 1)\pi$ to realize the total phase difference $\Delta \varphi = \Delta \varphi _a + \Delta \varphi _b = 2N\pi$; here, *m*, *n* and *N* are integers.

**Fig. 2:**
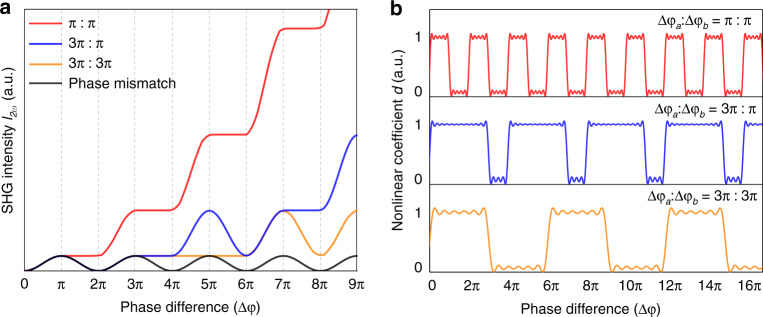
APP phase matching with light phase manipulation for nonlinear frequency conversion. **a** SHG intensity (*I*_*2ω*_) under phase mismatch and different APP phase-matching conditions with phase difference $\Delta \varphi$ in a nonlinear crystal. The relative SHG intensities with light phase manipulation of $\Delta \varphi _a:\Delta \varphi _b = \pi :\pi ,3\pi :\pi$ and $3\pi :3\pi$ correspond to the red, blue and orange lines, respectively. **b** Distribution of the second-order nonlinear coefficient *d* with the phase difference $\Delta \varphi$

We take the SHG process ($\omega + \omega = 2\omega$) as an example to elaborate APP phase matching. Considering the phase manipulation of $\Delta \varphi _a = \Delta \varphi _b = \pi$, the integral form of Eq. (2) is expressed as

4 $$E_{2\omega }\left( \varphi \right) = \frac{{2i\omega }}{{cn_{2\omega }}}E_\omega ^2\left[ {{\int_0^{\uppi}} {d_ae^{ - i\Delta \varphi }d\varphi } + {\int_{\uppi}^{2{\uppi}}} {d_be^{ - i\Delta \varphi }d\varphi } + {\int_{2{\uppi}}^{3{\uppi}}} {d_ae^{ - \Delta i\varphi }d\varphi + \cdots } } \right]$$

where in the interaction part *L*_*a*_ with nonlinear coefficient $d_a$, SHG operates and phase mismatch appears, while part *L*_*b*_ with nonlinear coefficient $d_b$ is used for manipulation of light phases, and the phase mismatch is compensated. Repeatedly, the SH intensity is enhanced with the phase difference $\Delta \varphi = 2\pi$. The relative SHG intensities (*I*_*2ω*_) with $\Delta \varphi _a = \Delta \varphi _b = \pi$, $\frac{1}{3}\Delta \varphi _a = \Delta \varphi _b = \pi$ and $\Delta \varphi _a = \Delta \varphi _b = 3\pi$ are shown in Fig. 2a, corresponding to the red, blue and orange lines, respectively. Ideally, the conversion efficiency of the situation corresponding to π-phase manipulation is the highest for the same interaction length.

As shown in Eq. (4), the intensities of SHG wavelengths are affected not only by the phase manipulation but also by the nonlinear coefficients of the APP structure. Under ideal conditions, the nonlinear coefficient is $d_b = 0$ in amorphous regions. If light propagates along the direction of the phase grating, then the distribution of second-order nonlinear coefficients with $\Delta \varphi _a:\Delta \varphi _b = \pi :\pi ,3\pi :\pi$ and $3\pi :3\pi$ is shown in Fig. 2b. Considering the different nonlinear coefficients of the crystalline/amorphous alignments, the effective nonlinear coefficient $d_{{{{\mathrm{eff}}}}}$ of the whole interaction process is given here. Associated with three-dimensional (3D) nonlinear photonics theory^21,27^, the APP structure can be modelled as a convolution of the unit function ${{u}}({\vec{r}})$ and the nonlinear coefficient distribution function $s(\vec r)$. Therefore, for APP phase manipulation condition of $\Delta \varphi = \Delta \varphi _a + \Delta \varphi _b = \left( {2m - 1} \right)\pi + \left( {2n - 1} \right)\pi = 2N\pi$, the nonlinear susceptibility in the 3D APP structure can be expressed as

5 $$\chi ^{{{{\mathrm{(2)}}}}}\left( {\vec{r}} \right){{{\mathrm{ = rect}}}}\left( {\frac{x}{X}} \right){{{\mathrm{rect}}}}\left( {\frac{y}{Y}} \right){{{\mathrm{rect}}}}\left( {\frac{z}{Z}} \right){{{\mathrm{ \times }}}}\left[ {u\left( {\vec{r}} \right) \otimes s\left( {\vec{r}} \right)} \right]$$

where the “rect” function is defined as $${{{\mathrm{rect}}}}\,(u) = \left\{ {\begin{array}{*{20}{c}} {1,} & {|u| \le \frac{{2m - {{{\mathrm{1}}}}}}{{2N}}} \\ {0,} & {{\mathrm{elsewhere}}} \end{array}} \right.$$ here, *m*, *n* and *N* are integers and X, Y and Z are the sizes of the 3D APP structure in three dimensions. According to the Fourier expansion of Eq. (5), the effective nonlinear coefficient can be written as

6 $$d_{{{{\mathrm{eff}}}}}\left( {f_x,f_y,f_z} \right) = {{{\mathrm{sinc}}}}\left[ {\pi {{{\mathrm{X}}}}\left( {f_x - mf_{ \wedge _x}} \right)} \right]{{{\mathrm{sinc}}}}\left[ {\pi {{{\mathrm{Y}}}}\left( {f_y - nf_{ \wedge _y}} \right)} \right]{{{\mathrm{sinc}}}}\left[ {\pi {{{\mathrm{Z}}}}\left( {f_x - qf_{ \wedge _z}} \right)} \right] \times {{{\mathrm{S}}}}\left( {{{\vec{f}}_{m,n,q}}} \right)$$

where the “sinc” function is defined as sinc (*x*) = sin(*x*)/*x*; ${\vec{f}}_{m,n,q} = mf_{ \wedge _x}{\hat{x}} + nf_{ \wedge _y}{\hat{y}} + qf_{ \wedge _z}{\hat{z}}$ and $f_{ \wedge _x} \wedge _x = f_{ \wedge _y} \wedge _y = f_{ \wedge _z} \wedge _z = 1$. $S({\vec{f}}_{m,n,q})$ is a geometric function calculated by

7 $${{{\mathrm{S}}}}\left( {\vec f_{m,n,q}} \right)=\,\frac{{\chi _a^{(2)} - \chi _b^{(2)}}}{V}{\int\!\!\!\!\!\int\!\!\!\!\!\int}{{{{\mathrm{exp}}}}\left( {i2\pi {\vec{f}}}_{m,n,q}{\vec{r}} \right)d^{3}r}$$

Here, the integration is taken over the APP unit structure with a volume of *V*, and $\chi _a^{(2)}$ and $\chi _b^{(2)}$ are the nonlinear susceptibilities of the crystalline/amorphous alignments, respectively.

**Fig. 3:**
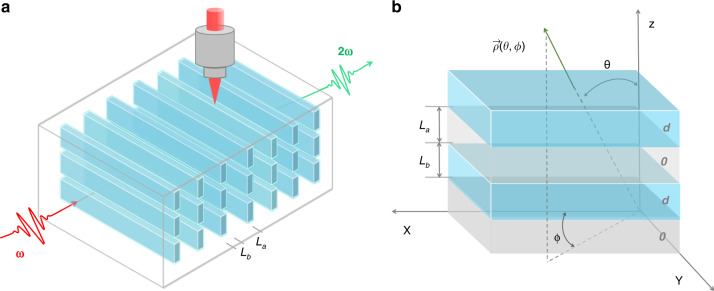
Angular APP phase-matching engineering diagram. **a** Schematic of laser direct writing of APP gratings. Multiple grating layers can be fabricated along the depth direction to obtain large-scale APP crystals. Λ = *L*_*a*_ *+* *L*_*b*_ is the fabricated APP grating period, and *L*_*a*_ and *L*_*b*_ are the lengths of crystalline/amorphous regions of the crystal with second-order nonlinear coefficients of *d*/0, respectively. **b** APP gratings along the Z direction. $\vec \rho \left( {\theta ,\phi } \right)$ is the propagation direction of the interacting waves; *θ* is the phase-matching angle between the propagation direction and optical axis; and $\phi$ is the azimuth angle

Next, we demonstrate the theoretical and experimental feasibility of angular engineering of the APP. If the light propagates in the direction of the phase grating, then a specific SHG wavelength can be obtained by special reciprocal vector compensation. In addition, new angular degrees of freedom in spatial modulation can be introduced with multiple phase grating layers fabricated along the depth direction to obtain large-scale APP crystals, which enables angular APP phase matching (Fig. 3a). In this way, we can realize tunable phase-matched SHG with angular engineering of the APP in one APP crystal. Compared with angular BPM limited by strict refractive index dispersion condition Δ*k* = 0 or angular QPM depending on ferroelectric materials^28–30^, the angular APP technique could realize tunable phase matching in the nonlinear optical materials no matter those with or without Δ*k* = 0 and reversed ferroelectric domains by continuous phase manipulation of Δ*φ* = 2*Nπ*. Moreover, angular APP strategy should be theoretically suitable for all non-centrosymmetric nonlinear crystals in the entire spectral transmission range. What’s more, large nonlinear coefficients could be employed under APP phase matching condition, which should be more flexible compared to the birefringence phase-matching condition.

Taking the structure of APP gratings arranged along the *z*-axis as an example, the interacting waves propagate along the direction of $\vec \rho \left( {\theta ,\phi } \right)$, where *θ* is the phase-matching angle between the propagation direction and *z*-axis and $\phi$ is the azimuth angle (Fig. 3b). Besides the changing of grating period length, the function of angle tuning is realized by the manipulation of the optical path to satisfy the phase relation of $\Delta \varphi = \Delta \varphi _a + \Delta \varphi _b = 2N\pi$ of periodic crystalline/amorphous regions by continuous phase compensations. In the crystalline regions, nonlinear frequency conversion proceeds with the phase difference $\Delta \varphi _a\left( {\theta ,\phi } \right) = (k_3\left( {\theta ,\phi } \right) - k_2\left( {\theta ,\phi } \right) - k_1\left( {\theta ,\phi } \right))z = \Delta {{k}}\left( {\theta ,\phi } \right)\frac{{L_a}}{{{\mathrm{cos}}\theta }}$. Associated with the APP phase matching, $\Delta \varphi _a\left( {\theta ,\phi } \right) = (2m - 1)\pi$ is required for efficient frequency conversion. For interacting waves with wave-vectors of $k_l\left( {\theta ,\phi } \right)$ (*l* = 1,2,3), there are 2^3^ types of polarization configurations for angular APP phase matching owing to the two different values of refractive indices *n*_*o*_ and *n*_*e*_, where *n*_*o*_ and *n*_*e*_ stand for the refractive indices of ordinary (o) light and extraordinary (e) light, respectively. Generally, a particular phase angle satisfies typical SHG light with a specific polarization; thus, the eight types of phase-matching conditions cannot coexist for the dispersion of intrinsic refractive indices. Due to the finite processing accuracy, the phase grating period is uncertainly distributed with an error of $L_r$; thus, an uncertain phase variation $\Delta \varphi _r$ is generated that participates in the phase compensation process, providing the possibility for tunable phase-matched SHG with different polarization configurations. Therefore, SHG is concurrently accessible for different types of angular APP phase matching, which greatly utilizes the pump light to improve the conversion efficiency. In the amorphous regions, phase compensation operates with $\Delta \varphi _b\left( {\theta ,\phi } \right) = 2\pi \left( {\frac{{n_3}}{{\lambda _3}} - \frac{{n_2}}{{\lambda _2}} - \frac{{n_1}}{{\lambda _1}}} \right)\frac{{L_b}}{{{\mathrm{cos}}\theta }}$, where *n*_*i*_ (*i* = 1,2,3) are the wavelength-dependent refractive indices of interacting waves, regardless of angle. For APP phase matching, $\Delta \varphi _b\left( {\theta ,\phi } \right) = (2n - 1)\pi$ from amorphous regions is added to compensate for the phase mismatch in the crystalline regions to realize effective energy conversion. Notably, in APP crystals, the refractive index of the material also varies periodically from the crystalline region to the amorphous region; however, the change in refractive index is very small (<0.005), so the influence on the refraction at the interface of the crystalline/amorphous regions can be ignored^26^.

We experimentally realized efficient tunable SHG in the UV spectral range with APP quartz, which has been identified as an efficient deep-UV nonlinear optical crystal by APP techniques^26^. A fibre femtosecond laser with a central wavelength of 1030 nm and a pulse width of 240 fs was used as the writing source. The minimum focused diameter of the femtosecond laser corresponding to amorphous region can be controlled within 2 μm, basically satisfying fabrication requirements of APP periods. The grating error is measured generally to be <500 nm. The phase gratings are arranged along the optical axis of a Z-cut quartz crystal. By optimizing the incident energy of the writing laser, the crystallinity of the crystal is obviously destroyed and transformed into an amorphous state through laser irradiation, which can be confirmed by the significant reduction in the Raman resonance peaks (see Supplementary Information for details, Fig. S2). A 3 mm long APP quartz crystal with a period length of *L*_*a*_ = *L*_*b*_ = 2.1 μm for SHG of the laser at a wavelength of 484 nm was fabricated based on the dispersion equation^31^, corresponding to light phase manipulation of $\Delta \varphi _a = \Delta \varphi _b = \pi$.

**Fig. 4:**
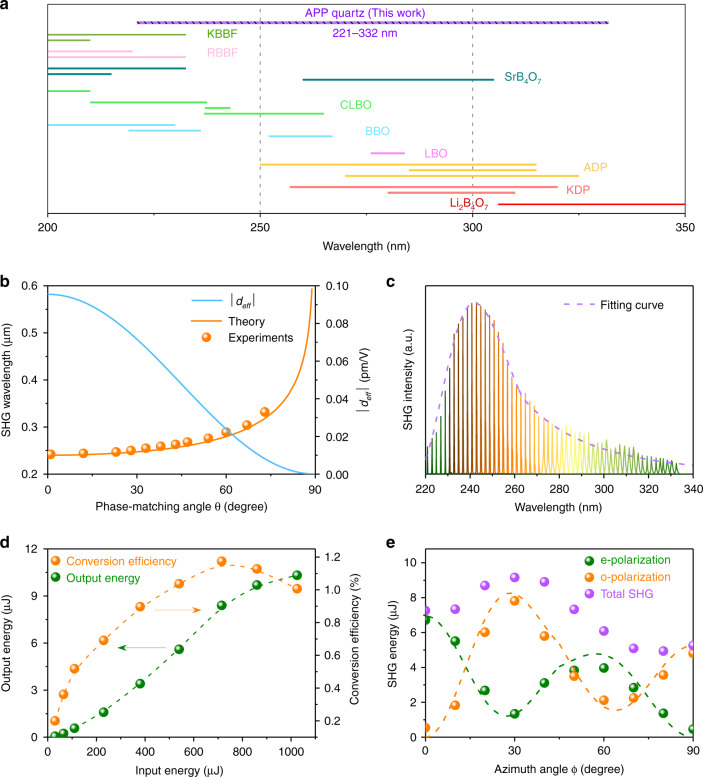
Experimental demonstration of angular APP phase matching with APP quartz with a period of $L_a = L_b = {\mathbf{2.1}}\,\upmu {\mathrm{m}}$. **a** Comparison of the tunable spectral region of this work with those of common nonlinear optical crystals in the deep-UV (200–350 nm) range. **b** Calculation of the angular APP phase-matching angle *θ* and distribution of absolute value |*d*_eff_| in the YZ plane when $\phi$ = 90°. The dots are the experimentally measured phase-matching angles corresponding to SHG wavelengths. **c** SHG signal intensities at different wavelengths from 221 to 332 nm. The dashed line is the fitting curve of SHG intensities. **d** Output energy and conversion efficiency of the SHG light at 242 nm. **e** Calculated (curves) and experimental (dots) SHG intensities for ordinary and extraordinary light at 250 nm with azimuth angle $\phi$ tuning from 0° to 90° along the Z direction, where the phase-matching angle is located at ${\theta}$ = 29.6°

An optical parametric oscillator (optical parametric oscillator, Opolette HE 355 II) with a pulse width of 10 ns and a repetition frequency of 20 Hz was used as the pump light source. First, angular APP phase matching was experimentally demonstrated in the YZ plane $(\phi = 90^ \circ )$ with extraordinary light incidence, corresponding to type (eeo) APP phase matching, and (o) and (e) stand for the ordinary and extraordinary polarizations, respectively. The relationship between the *d*_eff_ and incident angle $\left( {\theta ,\phi = 90^ \circ } \right)$ of APP quartz in the YZ plane calculated by Eq. (6) can be expressed as $d_{{\mathrm{eff}}} = \frac{1}{\pi }d_{11}{\mathrm{cos}}^2\theta {\mathrm{sin}}3\phi = - \frac{1}{\pi }d_{11}{\mathrm{cos}}^2\theta$. Widely tunable phase-matched SHG wavelengths from 221 to 332 nm were experimentally achieved through continuous phase-mismatch compensation by rotating the APP quartz along the X direction (Fig. 4a), which cover almost the entire UV range from 200 to 350 nm. The tunable SHG wavelengths are the results of the combination of angle tuning and phase variation participation. By theoretical analysis and fitting (orange line in Fig. 4b), the SHG wavelengths from 242 to 332 nm are found to be attributed to angular APP phase matching with phase-matched angle. In addition, the SHG wavelength from 221 to 241 nm is mainly attributed to the participation of phase variation $\Delta \varphi _r$ with a fixed angle, which is generated by the distribution of the entire grating period $\Lambda = L_a + L_b$ within a certain range from 3.8 to 4.6 μm with an error of *L*_*r*_ = ±0.4 μm. Associated with the statistical analysis of the phase variation $\Delta \varphi _r$ and angular engineering for $\Delta \varphi _a\left( {\theta ,\phi } \right)$ and $\Delta \varphi _b\left( {\theta ,\phi } \right)$, the experimental SHG signal intensities agree well with the theoretical calculations (purple dashed line in Fig. 4c). Therefore, the participation of phase variation $\Delta \varphi _r$ caused by fabrication error provides a realizable method for tunable phase matching. In fact, the designed APP grating structure and the accuracy of the grating fabrication will both affect the phase matching bandwidth. Besides, there is also another possible way for broadening the phase matching bandwidth by the introduction of the particular patterns (such as chirped periodic patterns, fan-out grating, segmented grating, etc.) into the APP materials for the realization of tunable SHG, which would be performed in the further experiments.

Compared with other common deep-UV nonlinear optical crystals, APP quartz shows a great advantage in wavelength tunability^32–49^ (Fig. 4a), regardless of BPM materials or QPM ferroelectric materials. The SHG energy reaches a maximum of 10.32 μJ under the input energy of 1025 μJ at a wavelength of 484 nm, corresponding to a SHG peak power of 1.46 kW and an optical conversion efficiency of *η* = 1.01% (Fig. 4d), which is almost 10 times higher than that of a QPM quartz with the finest twin structure (17.28 W, *η* = 0.12%) at 266 nm^50^. The conversion efficiency decreases slightly at high energy, which should be induced by the poor quality of the fundamental laser with the increase in the incident energy under the present conditions. The conversion efficiency can be enhanced by improving the laser fabrication accuracy and beam quality and peak power of fundamental light sources. In addition, the efficiency can also be improved with a nonlinear crystal with larger nonlinear coefficients and longer interaction length. Taking the BBO crystal as an example^47–49^, the realized tunable wavelength range of BBO crystal is about 20 nm in the ultraviolet range as shown in Fig. 4a. Moreover, the maximum output power as high as 1.37 W was recent realized at 213 nm with the efficiency over than 17% with a BBO crystal by the well-designed sum-frequency technique^18^, which indicates that the BBO crystal could be an excellent ultraviolet crystal and suitable for the high-power ultraviolet lasers with the APP strategy.

In addition, for realization of multiple types of APP phase matching, we also investigated the angular APP phase matching with a fixed phase-matching angle ${\theta}$ under arbitrary azimuth angle $\phi$. Taking the SHG process of pump light at a wavelength of 500 nm as an example, the phase-matching angles in the present APP quartz could be calculated to range from 25.64° to 35.40° for different types of APP phase matching; i.e., types {(ooe), (oee/eoe), (eee), (ooo), (oeo/eoo), (eeo)} correspond to {25.64°, 27.20°, 29.06°, 29.89°, 32.34°, 35.40°}, respectively. The incident angle was located at ${\theta}$ = 29.6° in the experiment with rotation of the APP quartz along the Z direction. Due to the contribution of the phase variation, all types of angular APP phase matching were observed in the SHG process. The SHG intensities for o light and e light were both measured in the experiment with azimuth angle $\phi$ tuning from 0° to 90° (Fig. 4e). By calculating the contribution of each type of phase matching, the experimental data (dots in Fig. 4e) agree well with the theoretical simulation (dashed curves in Fig. 4e). The total SHG energy reaches a maximum at an azimuth angle of approximately $\phi$ = 30°, and the output energy of SH light is 9.16 μJ with a fundamental wavelength energy of 1012 μJ, corresponding to o light and e light of 7.82 and 1.34 μJ, respectively. Therefore, the angular engineering by tuning azimuth angle *ϕ* can be employed not only to select nonlinear coefficients, but also to control the polarization output in different phase matching types, which should be very difficult or unavailable in traditional phase-matching conditions due to the dispersion of refractive index, thus could be further applied in relevant scientific fields, such as laser-induced periodic surface structures, laser-scanning nonlinear optical techniques and even some quantum entanglement fields with the nonlinear upconversion process^51–53^.

## Discussion

In conclusion, we have demonstrated angular engineering of an APP for widely tunable phase-matched SHG and experimentally demonstrated it in an APP quartz crystal. Through the introduction of angular engineering, efficient SHG was realized in an APP quartz with a tunability of 111 nm in the UV range and all possible phase-matching types were simultaneously realized. The optical conversion efficiency and peak power are over 1% and 1 kW, respectively, which could be further improved by improving the fabrication techniques and experimental conditions. Angular APP phase matching should provide a new route for nonlinear optics and thus will be key to opening the door for realization of tunable lasers, which could have applications in modern photonics and physics.

## Materials and methods

The quartz was cut along *z*-axis with the length of about 5 mm. The APP quartz with phase grating period of *L*_*a*_ *=* *L*_*b*_ *=* 2.1 μm was writing with the energy of 12 μJ by the femtosecond laser (ANTAUS 1030-20) with a pulse duration of 240 fs and a repetition rate of 200 kHz. The femtosecond laser with a central wavelength of 1030 nm was focused inside the crystal through a microscope objective (Mitutoyo 20x, NA = 0.35) into the sample, and the crystal is placed on a three-dimensional moving platform with the writing speed of 1 mm s^−1^. In order to obtain a large-scale APP quartz to meet angular manipulation requirements, five layers phase gratings were fabricated along the depth direction with an interlayer distance of 0.25 mm.

## Supplementary information

Supplementary information
