# Supplementary material for: Angular engineering strategy of an additional periodic phase for widely tunable phase-matched deep-ultraviolet second harmonic generation
Source: Light Sci Appl. 2022 Feb 4;11:31. doi: 10.1038/s41377-022-00715-w (PMC8816935; doi:10.1038/s41377-022-00715-w)
Supplement: Supplementary file 1 — Supplementary information [file 41377_2022_715_MOESM1_ESM.docx]

Supplementary Information for

**Angular engineering strategy of an additional periodic phase for widely tunable phase-matched deep-ultraviolet second harmonic generation**

Mingchuan Shao^1^, Fei Liang^1^, Haohai Yu^1^, Huaijin Zhang^1^

^1^*State Key Laboratory of Crystal Materials and Institute of Crystal Materials, Shandong University, Jinan 250100, China*

*Correspondence: Haohai Yu (haohaiyu@sdu.edu.cn) or Huaijin Zhang ([huaijinzhang@sdu.edu.cn](mailto:huaijinzhang@sdu.edu.cn))

1. **APP quartz sample fabrication**

In order to test the influence of different writing energies on optical properties of APP crystals, samples with the period of $\frac{1}{3}L_{a}=L_{b}=2.1 \mu m$ were also fabricated with different writing energy gradients, corresponding to the light phase manipulation of $\frac{1}{3}{\Delta\varphi}_{a}={\Delta\varphi}_{b}=\pi$ of the fundamental light at 484 nm (Fig. S1). Fig. S1a shows schematic diagram of femtosecond laser direct writing of APP gratings. The femtosecond laser is incident into the crystal after beam shaping. Fig. S1b, c, d shows the microscopic images of APP quartz samples, corresponding to writing energies of of 8 μJ, 12 μJ and 16 μJ, respectively. Although the writing energies are different, the grating period is well-distributed by optimizing fabrication parameters.

**
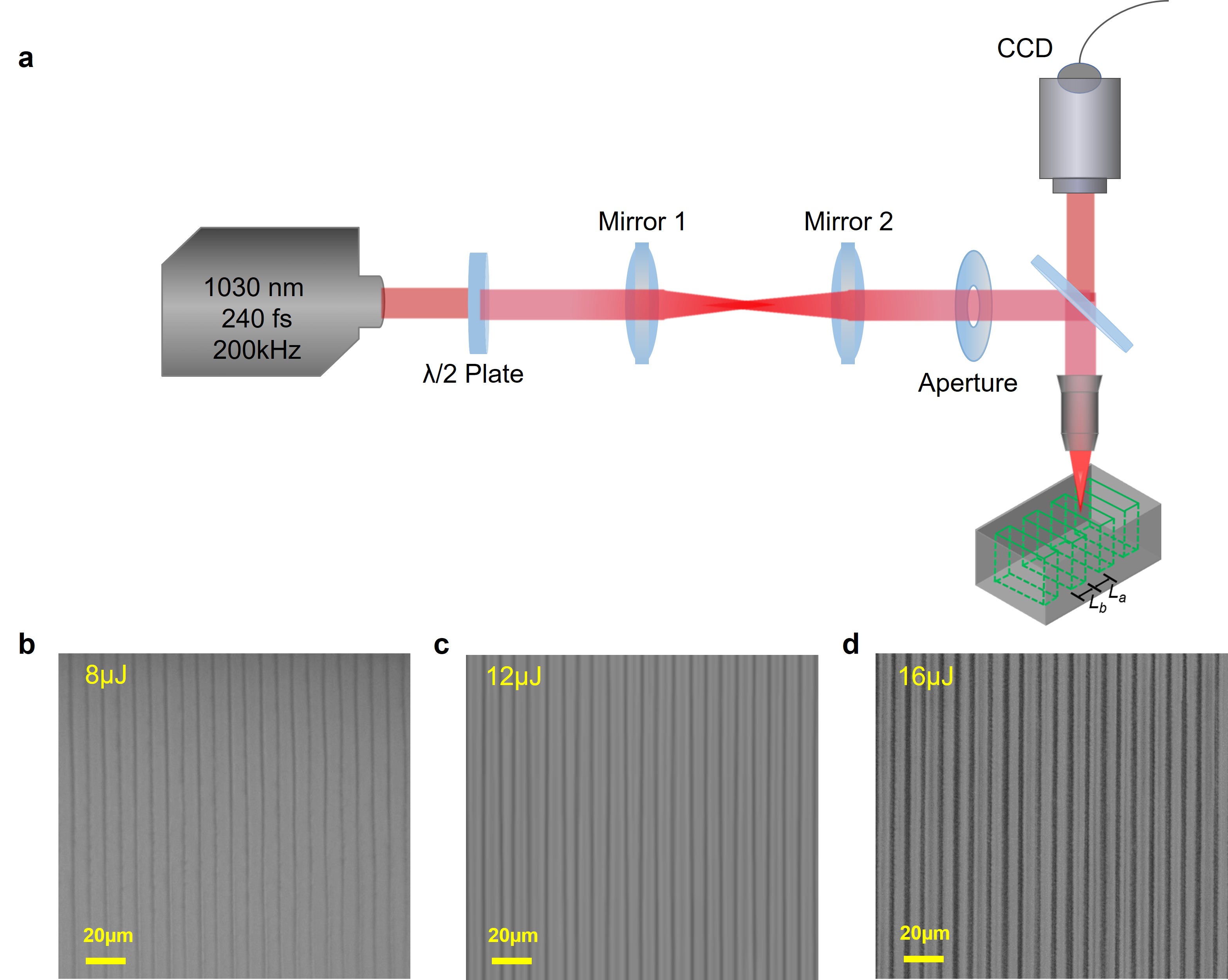
Fig. S1** Femtosecond laser direct writing phase gratings with different energies. (a) Schematic diagram of femtosecond laser direct writing phase gratings. Optical microscope images of phase gratings with the period of $\frac{1}{3}L_{a}=L_{b}=2.1 \mu m$ with different writing energies of 8 μJ (b), 12 μJ (c) and 16 μJ (d), respectively.

1. **Raman characterization of the APP quartz**

**

**

**Fig. S2** Comparison of Raman intensities under different writing energies. Black line shows the Raman intensity of pure quartz. The red, blue and orange lines correspond to Raman intensities of APP samples with the writing energies of 8 μJ, 12 μJ and 16 μJ, respectively.

For angular APP phase matching, the crystalline regions work for nonlinear frequency conversion and the amorphous regions provide phase compensation. Ideally, the effective nonlinear coefficient of laser writing region is *d_eff_* =0 for the damage of translational symmetry of the nonlinear crystals. In order to characterize the structural damage, we measured the Raman spectra of the APP samples under different writing energies. We can see a clear reduction of Raman peak intensity of the lithography area as the writing energy increases, indicating that femtosecond laser writing effectively reduces the vibration of molecular and a disordered region is induced^1^ (Fig. S2).

1. **SH image of APP sample in the YZ plane**

**
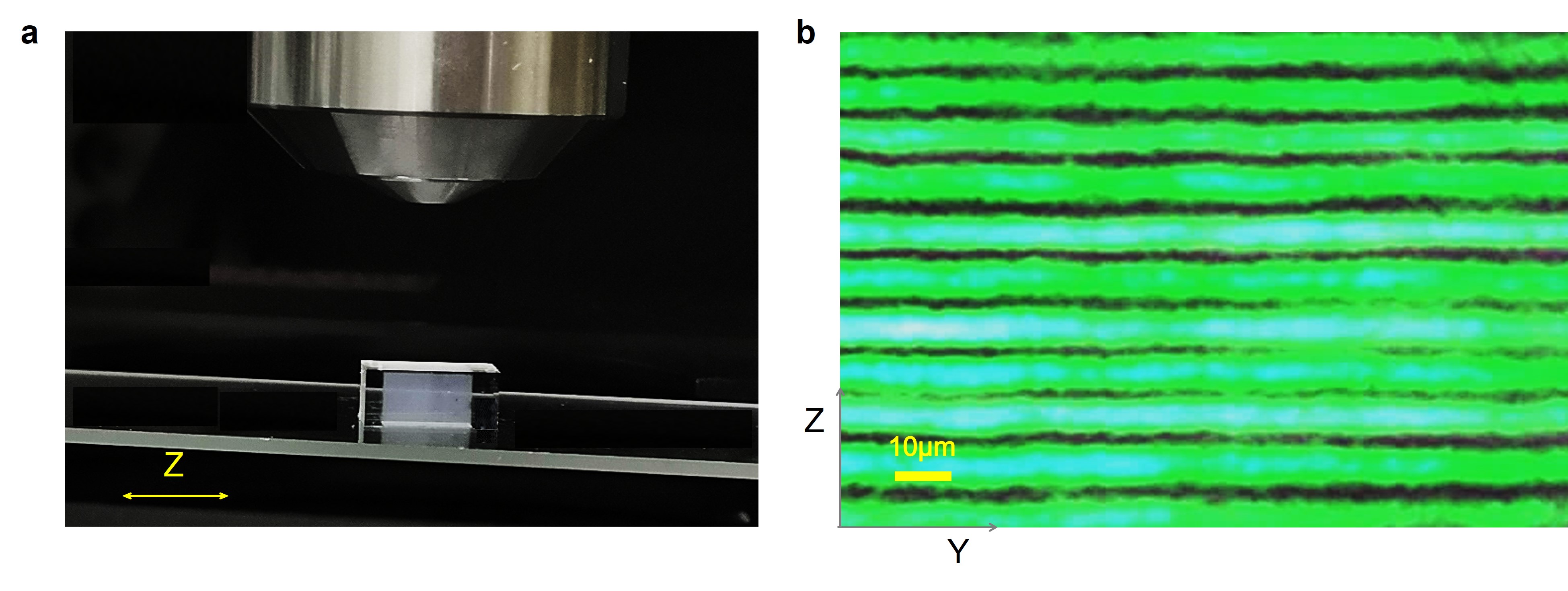
**

**Fig. S3** Images of APP quartz sample $\frac{\text{1}}{\text{3}}\text{L}_{\text{a}}\text{=}\text{L}_{\text{b}}\text{=2.1}\text{ }\text{μm}$ with the writing energy of 12 μJ. (a) APP quartz sample with a number of grating periods of 500 fabricated along Z direction. (b) SH image in the YZ plane of the crystalline regions (green parts) and amorphous regions (dark parts) through a general SH microscopic system.

Figure S3a shows the APP quartz sample fabricated with the period of $\frac{\text{1}}{\text{3}}\text{L}_{\text{a}}\text{=}\text{L}_{\text{b}}\text{=}\text{2.1}\text{ }\text{μm}$ under the writing energy of 12 μJ. The number of fabricated APP gratings is 500, corresponding to the effective APP quartz length of about 4.2 mm. We can see a clear nonlinear optical effect in crystalline regions with green light generated by SHG of fundamental light at 1030 nm under the SH confocal microscopy (Fig. S3b). The amorphous regions stay dark without second harmonic generation, indicating that there is no nonlinear optical effect in the laser writing areas^2,3^, whereas the dispersion of the refractive indices still exists, and the phase difference of the interacting lights could also be realized. We also observed an uncertain grating period variation $L_{r}$ at the interfaces of crystalline/amorphous regions, which provides the possibility for SHG with different polarization configurations with participation of phase variation $\Delta\varphi_{r}$.

1. **Optical loss of the APP grating structure**

Efficient frequency conversion needs to consider the effects of both absorption and gain. Figure S4 shows the optical loss of APP quartz under different writing energies with the unit of dB cm^-1^. The red, blue, orange and black lines correspond to writing energy intensities of 8 μJ, 12 μJ, 16 μJ and pure quartz, respectively. With the increase of writing energies, the loss of structure increases obviously, especially in the ultraviolet region. The increased loss of structure can be attributed to reflection and refraction loss due to the change of refractive indices of APP structure. Therefore, realization of a higher conversion efficiency can be expected by reducing loss of structure with appropriate writing energy.

**

**

**Fig. S4** Optical loss of phase grating structure of APP quartz for sample ${\frac{1}{3}L}_{a}=L_{b}=2.1 \mu m$ under different writing energies of 8 μJ (red line), 12 μJ (blue line) and 16 μJ (orange line), respectively.

**5. SHG effective coefficients of APP quartz**

The nonlinear coefficient of the quartz crystal and the phase-matching angles $\left( \theta,\phi\right)$ determine the *d_eff_* values. The *d_eff_* for all eight types of SHG conditions corresponding to phase difference $\Delta\varphi=\Delta\varphi_{a}+\Delta\varphi_{b}=\left( 2m-1 \right)\pi+\left( 2n-1 \right)\pi=2N\pi$ are shown in Table S1, here, *m*, *n* and *N* are integers. There is $d_{14}=-d_{14}=0$ by considering the Kleinman symmetry^4^. Although the effective nonlinear coefficient might be lower than that in birefringent phase-matching (BPM) only if it can be realized, the conversion efficiency could be comparable to that of BPM due to the joint contribution of the various types of APP phase matching and the efficient light-matter interaction length.

**Table S1.** SHG effective nonlinear coefficients of APP quartz with phase manipulation $\Delta\varphi=\Delta\varphi_{a}+\Delta\varphi_{b}=2N\pi$, corresponding to all the possible polarization configurations for angular APP phase matching. (o) and (e) stand for the ordinary and extraordinary polarizations, respectively. Considering the Kleinman symmetry, $d_{14}=-d_{14}=0$.

| Type  (ω+ω=2ω) | $d_{eff}^{(2)}$ |
| --- | --- |
| ooo | ${-\frac{1}{N\pi}d}_{11}$sin3$\phi$ |
| eee | ${-\frac{1}{N\pi}d}_{11}\cos^{3}\theta$cos3$\phi$ |
| ooe | ${\frac{1}{N\pi}d}_{11}$cos*θ*cos3$\phi$ |
| eeo | ${\frac{1}{N\pi}[d}_{11}\cos^{2}\theta$sin3$\phi$-$d_{14}$sin2*θ*] |
| oeo/eoo | ${\frac{1}{N\pi}d}_{11}$cos*θ*cos3$\phi$ |
| oee/eoe | ${\frac{1}{N\pi}[d}_{11}\cos^{2}\theta$sin3$\phi$+$\frac{1}{2}d_{14}$sin2*θ*] |

**6.** **Acceptance angle of APP quartz**

Based on nonlinear optical theory, the acceptance angle determines the achievable wavelength range under the phase-matching conditions. We measured the acceptance angle at the fundamental wavelength of 484 nm for type (ooo) APP phase matching with the results shown in Fig. S5. The acceptance angle is about 5.9° comparable with the birefringence phase matching (BPM) and quasi-phase matching (QPM) with the acceptance angle of generally less than 5°^5^.





**Fig. S5** Experiments of SHG intensity versus the internal angle for angular APP phase matching corresponding to frequency conversion from 484 nm to 242 nm.

**7. Temperature-dependent tunable SHG experiment of APP quartz**

We have also experimentally demonstrated the influence of temperature on phase-matched wavelength. Under the certain temperature *T*, the phase difference in a single section can be expressed as

$\Delta\varphi(T)=\Delta k\left( T \right)\Lambda\left( T \right)=\Lambda\left( T \right)[{(k}_{2\omega}\left( T \right)-2k_{\omega}\left( T \right)]-2m\pi$ (S1)

where m is the order of phase matching and Λ(T) is the grating period length. It is observed that $\Delta k\left( T \right)$ will be affected by thermal refractive index coefficients dn/dT and thermal expansion coefficients α of crystals concurrently.

For a z-cut quartz crystal, the thermal expansion coefficient α is about 0.55*10^-6^/℃^6^ for amorphous region and 7.5*10^-6^/℃^7^ for crystalline region. The thermal refractive index coefficients are expressed as^8^

$$\frac{dn_{o}}{dT}=(-3.8572\lambda^{3}+7.0195\lambda^{2}-4.5236\lambda+0.4840)\times{10}^{-5}$$

$\frac{dn_{e}}{dT}=(-7.6314\lambda^{3}+13.0119\lambda^{2}-7.6193\lambda+0.8941)\times{10}^{-5}$ (S2)

Which are listed in Table S2 at different wavelengths.

**Table S2.** Thermal refractive index coefficients of quartz crystal for different wavelengths.

| λ/μm | 0.441 | 0.467 | 0.480 | 0.508 | 0.589 | 0.643 |
| --- | --- | --- | --- | --- | --- | --- |
| dn_o_/dT(10^-5^/℃) | -0.476 | -0.490 | -0.497 | -0.508 | -0.533 | -0.548 |
| dn_e_/dT(10^-5^/℃) | -0.590 | -0.603 | -0.609 | -0.619 | -0.639 | -0.654 |

Figure S6 shows the calculation (blue line) and experiment (blue dots) of temperature-dependent phase-matched SHG wavelengths of APP quartz. It is found that the phase-matched SHG wavelength remains almost unchanged with the change of temperature, which indicates that the change of temperature on phase matching is not large enough to obviously affect the phase matching wavelength, especially for the short wavelength with large phase difference in the ultraviolet region.

**

**

**Fig. S6** Calculation (blue line) and experiment (blue dots) of temperature-dependent phase-matched SHG wavelengths of APP quartz.

**References**

1. Matsuo, S. *et al*. Femtosecond laser assisted etching of quartz: microstructuring from inside. *Applied Physics A* **84**, 99-102 (2006).
2. Xu, T. X. *et al.* Three-dimensional nonlinear photonic crystal in ferroelectric barium calcium titanate. *Nature Photonics* **12**, 591-595 (2018).
3. Wei, D. Z. *et al*. Experimental demonstration of a three-dimensional lithium niobate nonlinear photonic crystal. *Nature Photonics* **12**, 596-600 (2018).
4. Kleinman, D. A. Nonlinear dielectric polarization in optical media. *Physical Review* **126**, 1977-1979 (1962).
5. Wang, T. *et al*. Large angle acceptance of quasi-phase-matched second harmonic generation in a homocentrically poled LiNbO_3_. *Optics Communications* **252**, 397-401 (2005).
6. Jiro, O. & Tomisi, K. Thermal expansion of fused quartz. *Metrologia* **5**, 50-55 (1969).
7. Kosinski, J. A., Gualtieri, J. G. & Ballato, A. Thermal expansion of alpha quartz. Proceedings of the 45th Annual Symposium on Frequency Control, pp. 22-28 (1991).
8. Zhao, S. & Wu, F. Q. The study on dispersive equation and thermal refractive index coefficient of quartz crystal. Acta Photonica Sinica **35**, 1183-1186 (2006).
